# Schisandrin B Attenuates Airway Inflammation by Regulating the NF-*κ*B/Nrf2 Signaling Pathway in Mouse Models of Asthma

**DOI:** 10.1155/2021/8029963

**Published:** 2021-06-28

**Authors:** Yaqin Chen, Yu Kong, Qili Wang, Jian Chen, Hua Chen, Huihui Xie, Lan Li

**Affiliations:** ^1^Department of Pediatrics, The First Affiliated Hospital of Zhejiang Chinese Medical University, Hangzhou, Zhejiang 310006, China; ^2^The First Clinical Medical School, Zhejiang Chinese Medical University, Hangzhou, Zhejiang 310006, China; ^3^Tricare Management Activity, Zhejiang Chinese Medical University, Hangzhou, Zhejiang 310006, China

## Abstract

**Background:**

Asthma is a complex inflammatory disorder that plagues a large number of people. Schisandrin B is an active ingredient of the traditional Chinese herbal medicine Schisandra with various proven physiological activities such as anti-inflammatory and antioxidant activities. In this study, we explored the anti-inflammatory and antioxidant effects and provided the mechanistic insights into the activity of schisandrin B in a mouse model of ovalbumin- (OVA-) induced allergic asthma.

**Methods:**

Male BALB/c mice were sensitized and challenged with OVA to induce asthma and treated with various doses (15 mg/kg, 30 mg/kg, and 60 mg/kg) of SCH to alleviate the features of allergic asthma, airway hyperresponsiveness, inflammatory response, OVA-specific immunoglobulin (Ig)E level, and pathological injury.

**Results:**

Schisandrin B significantly attenuated the airway hyperresponsiveness induced by OVA. Moreover, schisandrin B administration suppressed inflammatory responses, reduced the level of IgE, and attenuated pathological injury. Mechanistically, schisandrin B treatment promoted the activation of nuclear erythroid 2-related factor 2 (Nrf2), but suppressed the stimulation of the NF-*κ*B pathway caused by OVA.

**Conclusion:**

Taken together, our study suggests that schisandrin B attenuates the features of asthmatic lungs by inhibiting the NF-*κ*B pathway and activating the Nrf2 signaling pathway.

## 1. Introduction

Asthma is a chronic and complicated airway disease that has become a global health problem in recent years. Approximately one-fifth of the current total global population has been diagnosed with asthma, and the prevalence, morbidity, and mortality have rapidly increased in the past few years [1, 2]. Although several patients are well controlled by therapy such as corticosteroid inhalation, severe symptoms are difficult to control. Therefore, an understanding of the precise pathology and the search for perfect treatment methods are important to achieve a better management of asthma.

The classic manifestation of bronchial asthma is chronic airway inflammation, which is distinguished by airway inflammation dominated by eosinophils, increased mucus production, and airway hyperresponsiveness (AHR) [3]. Interleukin-4 (IL-4), IL-13, and IL-5 are type 2 (Th2) cytokines that support the vital role of Th2 lymphocytes in asthma pathogenesis by reducing the release of immunoglobulin (Ig)E and eosinophil infiltration into lung tissues, which accelerate the process of allergic asthma [4, 5]. Meanwhile, the production of reactive oxygen species (ROS), catalase (CAT), glutathione peroxidase (GSH-Px), superoxide dismutase (SOD), nitric oxide (NO), and malondialdehyde (MDA) imbalanced in an asthma model compared with a normal model [6]. Moreover, a large number of signaling pathways are involved in the pathophysiology of asthma. Both nuclear factor kappa-light-chain-enhancer of activated B cells (NF-*κ*B) and nuclear erythroid 2-related factor 2 (Nrf2) pathways are strongly linked to airway inflammation and remodeling in asthma [7]. Overstimulation of the NF-*κ*B pathway regulates the expression of airway inflammatory factors and their excessive secretion from inflammatory cells in allergic asthma [5]. Nrf2 is a main regulator of oxidative stress responses. Specific activation of the Nrf2 pathway enhances the inhibition of the airway inflammatory response and significantly reduces allergen-induced AHR, secretion of mucus, and discharge of Th2 cytokines (IL-13, IL-5, and IL-4) [8].

Schisandrin B (SCH), a lignan active ingredient, was extracted from Schisandra, a traditional Chinese herbal medicine. In addition, it exerts a wide range of effects on physiological activities, such as anti-inflammatory, antioxidant, and antitumor effects and liver toxicity [9]. A previous study reported that the water extract of Schisandra has a certain role in protecting against lung injury, which may be related to improving oxidative stress and airway inflammation, mainly manifesting as the inhibition of the infiltration of neutrophils and macrophages in lung tissues, the production of NO, and the secretion of IL-8 and MCP-1 [10]. SCH restrains the LPS-induced excessive inflammatory response in human umbilical vein endothelial cells by activating the Nrf2 pathway [11]. Moreover, SCH also attenuates airway damage and inflammation-induced lung injury caused by the inhalation of cigarettes by restricting the NF-*κ*B pathway and activating the Nrf2 pathway [11]. Nevertheless, the protective effect and potential mechanism of SCH in asthma remain unclear. This study focused on the antiasthmatic effect of SCH and its theoretical foundation in asthmatic models. We aimed to explore the potential protective role of SCH in asthma through the activation of Nrf2 and inhibition of the NF-*κ*B pathway.

## 2. Materials and Methods

### 2.1. Animals

Specific pathogen-free (SPF) inbred BALB/c mice aged approximately 6–8 weeks were purchased from Zhejiang Chinese Medical University Animal Experiment Center (Certificate No. SYXK (Zhe) 2018-0012) and under standard laboratory conditions at a constant temperature of 25°C and a 12-12 h day/night cycle for 7 days before the start of the experiments. The mice were freely provided water and standard food.

This study was authorized by the Animal Ethics Committee of Hangzhou Eyong Biotechnological Co., Ltd., and all experiments were conducted according to the Guidelines for the Care and Use of Laboratory Animals.

### 2.2. Experimental Mouse Model of Allergic Asthma

Sixty BALB/c male mice weighing 18-20 g were stochastically distributed into 6 groups (equal distribution): normal group (control), ovalbumin- (OVA-) induced asthma model group (OVA), SCH (Sigma-Aldrich, China) low-dose group (15 mg/kg, OVA+SCH-L), SCH middle-dose group (30 mg/kg, OVA+SCH-M), SCH megadose group (60 mg/kg, OVA+SCH-H), and positive control group (0.5 mg/kg, dexamethasone, OVA+DXM). Mice were sensitized several times by injecting a 2 mg/mL ovalbumin (OVA, Grade V) solution with 4% aluminum hydroxide gel (Sigma-Aldrich, China) to establish an asthma model. On the first day, 0.5 mL of the OVA solution was injected into the hind feet, groin, subcutaneous, cervical subcutaneous area, and abdominal cavity of each mouse [12]. Then, on the 14^th^ day, 0.2 mL of the OVA solution was injected into the abdominal cavity to strengthen the allergic response. Finally, on the 23^rd^ day, an aerosolized physiological saline solution with 10 mg/mL ovalbumin was inhaled by mice for 30 min, at a frequency of 3 times a week for 2 months. Along with OVA physiological saline, all mice were administrated SCH or normal saline by oral gavage 1 h before OVA challenge once a day for 2 months. Dexamethasone (Sigma-Aldrich, China) was administered to mice in the positive control group 3 times a week for 2 months.

### 2.3. The Evaluation of AHR

One day after the final OVA treatment, conscious mice were placed into the plethysmography chamber (EMKA, Germany) to record baseline readings for an evaluation of lung function. Then, the mice sequentially inhaled 50 *μ*L of normal saline and 0.0625, 0.125, 0.25, 0.5, 1.0, and 2.0 mg/mL aerosolized methacholine (Mch). Lung resistance (*R*_L_) and dynamic compliance (*C*_dyn_) were recorded at every stage.

### 2.4. Enzyme-Linked Immunosorbent Assay (ELISA)

ELISA kits (R&D Systems, USA) were employed to detect the level of the inflammatory factors eotaxin, IL-13, IL-5, and IL-4 in BALF according to the manufacturer's instructions. Moreover, OVA-specific IgE in the blood samples was also detected by using ELISA kits (BioLegend, USA) according to the manufacturer's instructions. The antibodies used were purchased from Abcam.

### 2.5. Analysis of Inflammatory Cells in Bronchoalveolar Lavage Fluid (BALF)

One day after the final OVA stimulation, the mice were sacrificed by anesthesia using a 0.75% pentobarbital solution. Then, the trachea was cannulated with a deeply inserted catheter and the left upper lobe was ligated. The lungs were lavaged for three times to collect bronchoalveolar lavage fluid using 700 *μ*L of cold PBS, and the lung tissues were collected. BALF cells were centrifuged with the cytospin technique and stained with Swiss Giemsa dye to count the total cells, lymphocytes, neutrophils, eosinophils, and macrophages.

### 2.6. Histopathological Analysis of the Lung

A 4% paraformaldehyde solution was used to fix the converged lung tissues of mice for 24 h. After embedding in paraffin, 4 *μ*m slices were cut and a light microscopy examination was performed after hematoxylin-eosin (H&E) and Alcian blue-periodic acid–Schiff (AB-PAS) staining. Inflammation of the airway was observed by assessing morphological changes in the lung tissue using H&E staining, while AB-PAS staining was carried out to determine goblet cell hyperplasia. The detailed methods are described in a previous study [13].

### 2.7. Immunohistochemistry

Lung slices with a thickness of 4 *μ*m were placed in a 60°C oven for 2 h. The slices were dewaxed with xylene, dehydrated with graded concentrations of alcohol, incubated in an extraction buffer, and incubated with a high-power microwave for 15 minutes. The slices were then cooled, washed with washing buffer, and sequentially incubated with a primary antibody against NF-*κ*B p65 (1 : 1000, Santa Cruz, CA, USA), Nrf2 (1 : 200, CST, USA), iNOS (1 : 200, Abcam, Cambridge, UK), or HO-1 (1 : 200, CST, USA) and secondary antibody (1 : 2000, CST, USA). Finally, the sections were stained with diaminobenzidine (DAB), counterstained with hematoxylin, cleared with xylene, and fixed. Five different microscope fields were randomly selected under a 200x magnification lens.

### 2.8. Western Blot

The levels of p-IKK*α*, IKK*α*, p-NF-*κ*B, NF-*κ*B, p-I*κ*B*α*, I*κ*B*α*, Nrf2, and HO-1 were determined to detect the levels of proteins related to the NF-*κ*B and Nrf2 pathways, with GAPDH serving as the internal standard. First, proteins were extracted from the collected lung tissues, and then, the total protein content was quantified using a bicinchoninic acid (BCA) protein detection kit (Beyotime, Hangzhou, China). Protein samples with the same concentrations were separated on 10% SDS-PAGE gels, and the proteins were transferred onto porous nitrocellulose (NC) membranes (Thermo Fisher Scientific, USA). Afterwards, the membranes were blocked with nonfat milk and incubated with the primary antibodies at 4°C for 24 h. The blots were incubated with the secondary goat anti-rabbit antibody at room temperature for 1 h. Blots were incubated with the ECL reagents (Beyotime Biotechnology, Shanghai, China) and analyzed using ImageJ software. All the antibodies used in this study were purchased from CST.

### 2.9. Real-Time Quantitative RT-PCR

Total RNA was isolated from the lungs using TRIzol reagent (Invitrogen, Carlsbad, CA, USA). The expression of the KC and GAPDH mRNAs was detected with real-time PCR using SYBR green (Takara), an ABI StepOnePlus real-time PCR system, and the following primers: KC forward 5′-CAATGAGCTGCGCTGTCAGTG-3′ and reverse 5′-CTTGGGGACACCTTTTAGCATC-3′; GAPDH forward 5′-GGGAAACCCATCACCATCTT-3′ and reverse 5′-CCAGTAGACTCCACGACATACT-3′. The thermocycling protocol was as follows: 95°C predenaturation for 10 min, followed by 40 cycles of 95°C for 5 s and 60°C for 1 min. GAPDH was chosen as the reference gene, and the formula (2^−*ΔΔ*Ct^) was used to the relative expression level of the tested genes.

### 2.10. Detection of Oxidative Stress in the Lung

Homogenates of the lung tissues with the same weight were prepared in 0.05 M Tris-HCl buffer (pH 7.4). The homogenate supernatant was collected to measure CAT, ROS, SOD, GSH-Px, MDA, and NO levels with commercial kits (Jiancheng, Nanjing, China) according to the manufacturer's protocol.

### 2.11. Statistical Analysis

Data are presented as the means ± standard deviations (SD). Values of *p* < 0.05 were accepted as statistically significant among different comparisons. One-way ANOVA and Tukey's multiple-comparison test were used to compare multiple groups.

## 3. Results

### 3.1. SCH Ameliorates AHR

**Figure 1:**
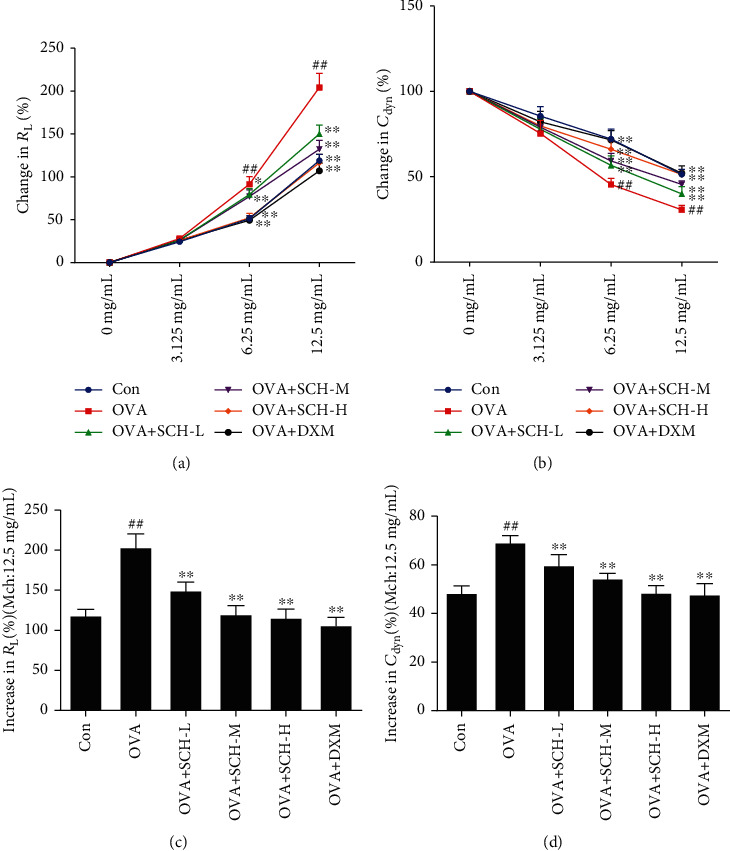
Effect of SCH on AHR. (a, b) *R*_L_ (%) and *C*_dyn_ (%) were evaluated. (c, d) *R*_L_ (%) and *C*_dyn_ (%) were again assessed separately after the administration of an Mch dose of 12.5 mg/mL. Data are reported as the means ± SD; *n* = 6. ^###^*p* < 0.001 and ^##^*p* < 0.01 compared with the control group; ^∗∗^*p* < 0.01 and ^∗^*p* < 0.05 compared with the OVA group.

Airway reactivity is evaluated by calculating the percentage of *C*_dyn_ [14], which is interpreted as the change in lung volume in response to pressure, and *R*_L_, which is the compression force driving breathing divided by flow [13]. OVA-induced mice received a series of doses of SCH to further determine the effect of SCH on asthmatic AHR. As shown in Figures 1(a) and 1(c), compared with the control, *C*_dyn_ of the asthma model mice was significantly reduced. However, this reduction was actually reversed by SCH treatment, and high concentrations of SCH produced better results. In addition, compared with the control, the lung resistance (*R*_L_) of the asthma model mice was significantly increased. As expected, the administration of dexamethasone decreased *R*_L_ in a dose-dependent manner (Figures 1(b) and 1(d)).

### 3.2. SCH Suppresses Inflammatory Cell Infiltrations in BALF

**Figure 2:**
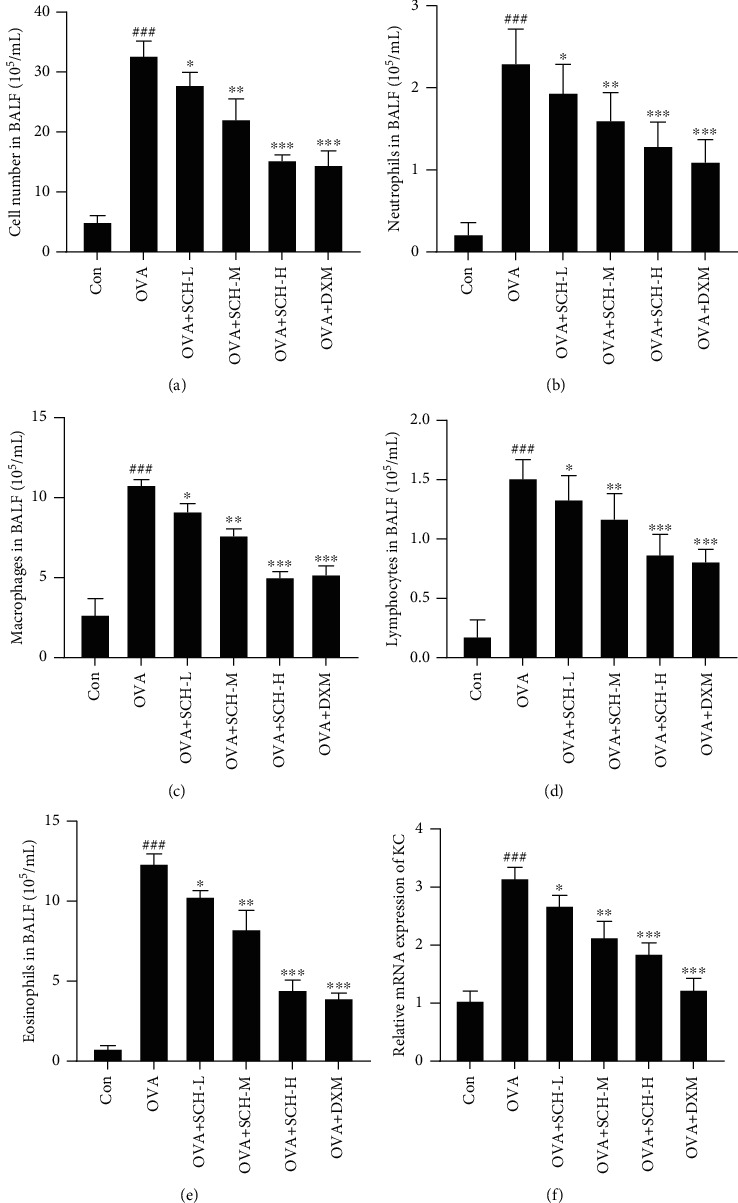
Effect of SCH on the number of inflammatory cells in BALF. The BALF was collected to determine the number of (a) total cells, (b) neutrophils, (c) lymphocytes, (d) macrophages, and (e) eosinophils and the (f) expression of the keratinocyte-derived protein chemokine (KC). Data are presented as the means ± SD; *n* = 6. ^###^*p* < 0.001 compared with the control group; ^∗∗∗^*p* < 0.001, ^∗∗^*p* < 0.01, and ^∗^*p* < 0.05 compared with the OVA group.

Inflammatory cells in the BALF were counted to determine the effectiveness of SCH at inhibiting the infiltration of inflammatory cells from the circulation into the lung space (BALF). The total cell number (Figure 2(a)) and counts of neutrophils (Figure 2(b)), lymphocytes (Figure 2(c)), macrophages (Figure 2(d)), and eosinophils (Figure 2(e)) in BALF were dramatically increased in asthmatic mice, while the increases were restrained by SCH in a dose-dependent manner. We also observed increased expression of the KC mRNA in asthmatic mice following the OVA intervention using qPCR, while this increase was inhibited by SCH in a dose-dependent manner (Figure 2(f)).

### 3.3. SCH Reduces the Airway Levels of Inflammatory Factors

**Figure 3:**
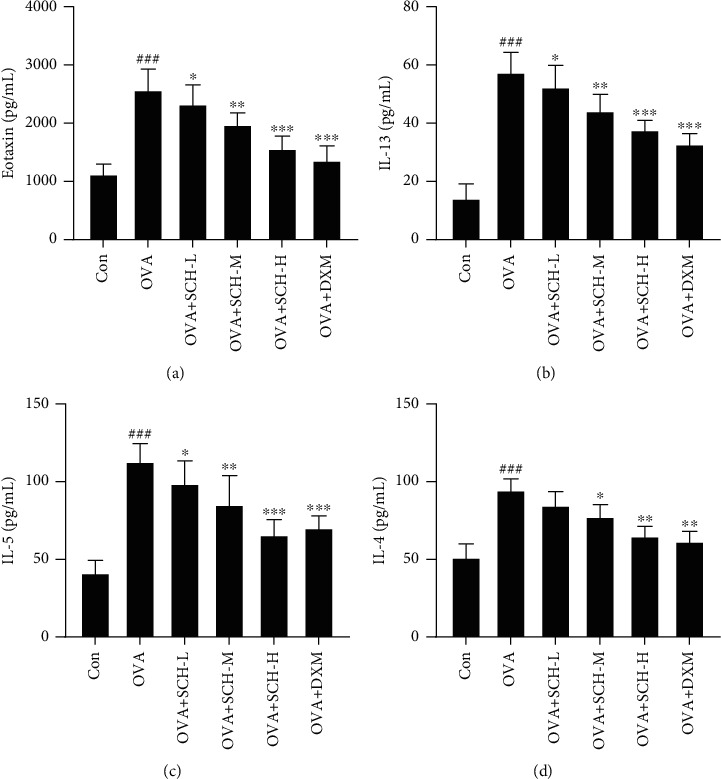
Effect of SCH on the airway levels of inflammatory factors. Samples of BALF were collected to detect the levels of (a) eotaxin, (b) IL-15, (c) IL-5, and (d) IL-4 via using ELISA. Data are reported as the means ± SD; *n* = 6. ^###^*p* < 0.001 and ^##^*p* < 0.01 compared with the control group; ^∗∗∗^*p* < 0.001, ^∗∗^*p* < 0.01, and ^∗^*p* < 0.05 compared with the OVA group.

Levels of the inflammatory factors eotaxin, IL-13, IL-5, and IL-4 were assessed using ELISAs to investigate whether SCH regulated the OVA-induced airway inflammatory response. As anticipated, SCH administration significantly reduced the OVA-induced increases in the levels of eotaxin (Figure 3(a)), IL-13 (Figure 3(b)), IL-5 (Figure 3(c)), and IL-4 (Figure 3(d)), and the high dosage of SCH exerted an obvious effect.

### 3.4. SCH Reduces the Blood Level of OVA-Specific IgE

**Figure 4:**
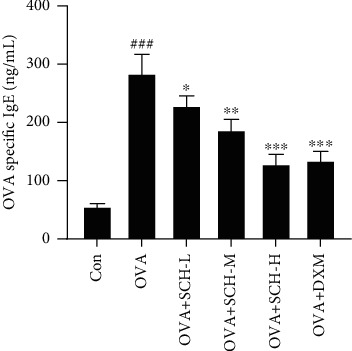
Effect of SCH on the level of OVA-specific IgE. BAL fluids were collected to detect OVA-specific IgE. Data are presented as the means ± SD; *n* = 6. ^###^*p* < 0.001 compared with the control group; ^∗∗∗^*p* < 0.001, ^∗∗^*p* < 0.01, and ^∗^*p* < 0.05 compared with the OVA group.

Next, OVA-induced asthmatic mice received a large dosage of SCH to determine whether the level of OVA-specific IgE was affected by SCH. As shown in Figure 4, the level of OVA-specific IgE was markedly higher in the asthmatic group than in the control group, while SCH reduced this level in a dose-dependent manner (Figure 4).

### 3.5. SCH Attenuates Histological Lung Injury

**Figure 5:**
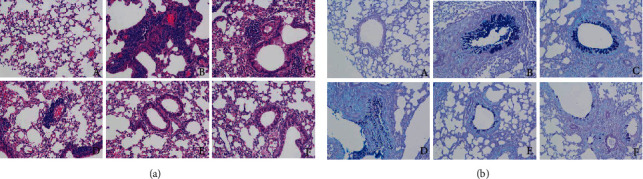
Effect of SCH on lung cell histology. (a) The lung tissues were combined to visualize the pathological condition using H&E staining. (b) Goblet cell hyperplasia in different groups was detected using AB-PAS staining. Magnification, ×200. Group notations: A: control group; B: OVA group; C: OVA+SCH-L group; D: OVA+SCH-M group; E: OVA+SCH-H group; F: OVA+DXM group.

Lung tissues were collected from different groups of mice for a histopathological study. Consistent with the results for the inflammatory response, H&E staining of lung tissues clearly indicated a significantly increased quantity of inflammatory cells in the asthma model group, while the SCH treatment reduced inflammatory cell infiltration in a dose-dependent manner (Figure 5(a)). AB-PAS staining was conducted on lung sections to assess the beneficial effects of SCH on cell hyperplasia. SCH administration significantly alleviated the OVA-induced secretion of mucus by goblet cells, and high dose of SCH exerted a better effect (Figure 5(b)).

### 3.6. SCH Alleviates Oxidative Stress in the Lung

**Figure 6:**
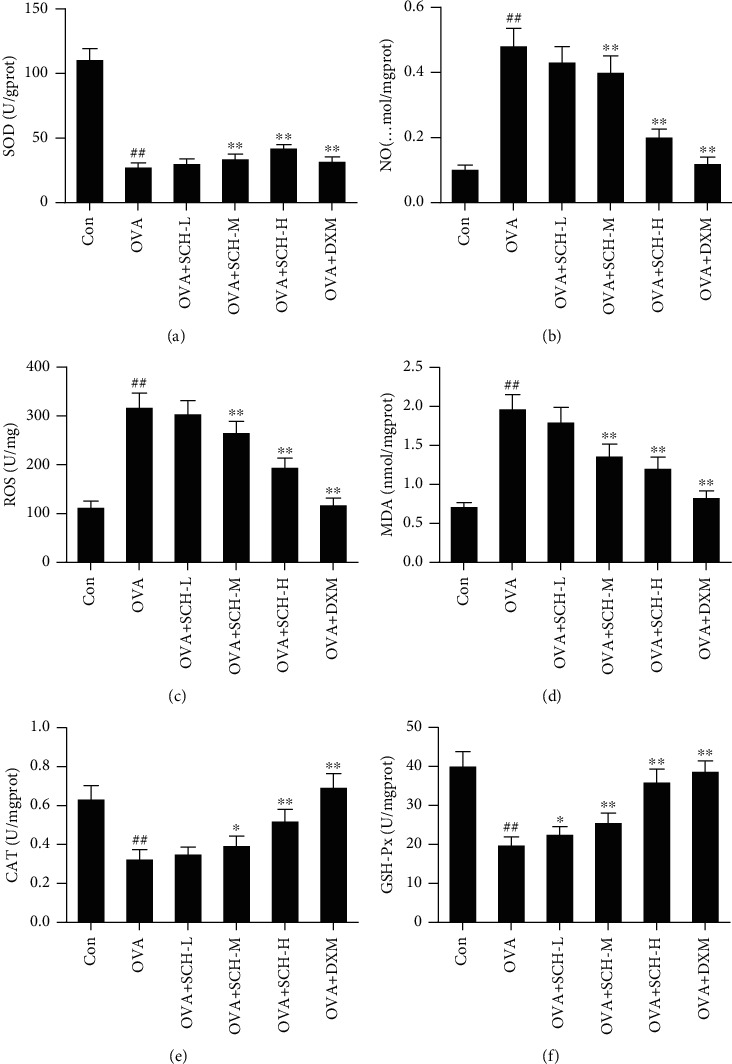
Antioxidant effects of SCH on the lung. The levels of (a) SOD, (b) NO, (c) ROS, (d) MDA, (e) CAT, and (f) GSH-Px in lung tissues were investigated to evaluate the antioxidant function of SCH. Data are presented as the means ± SD; *n* = 6. ^##^*p* < 0.01 compared with the control group; ^∗∗^*p* < 0.01 and ^∗^*p* < 0.05 compared with the OVA group. MDA: malondialdehyde; SOD: superoxide dismutase; ROS: reactive oxygen species; NO: nitric oxide; GSH-Px: glutathione peroxidase; CAT: catalase.

The expression of SOD, NO, ROS, MDA, CAT, and GSH-Px was detected to evaluate antioxidant activity in the lung. As shown in Figure 6(a), SOD levels were substantially decreased in the OVA group compared with the control group. In the OVA+SCH-M, OVA+SCH-H, and OVA+DXM groups, SOD levels were substantially increased compared with those in the OVA group. In contrast, the levels of ROS, NO, and MDA were substantially increased in the OVA group compared with the control group but greatly decreased in the OVA+SCH-M, OVA+SCH-H, and OVA+DXM groups compared with the OVA group (Figures 6(b)–6(d)). CAT and GSH-Px levels were substantially decreased by OVA-induced injury but noticeably increased by SCH and DXM compared with the OVA group (Figures 6(e) and 6(f)).

### 3.7. SCH Inhibits NF-*κ*B Pathways

**Figure 7:**
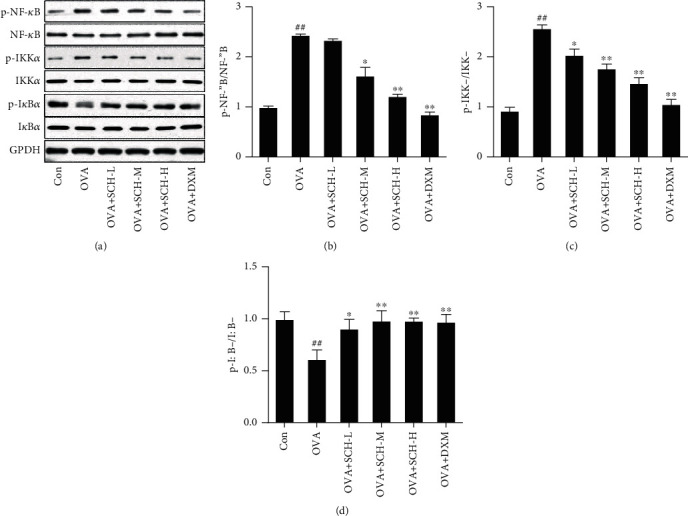
Effect of SCH on NF-*κ*B pathways. (a) The levels of p-NF-*κ*B, NF-*κ*B, p-IKK*α*, IKK*α*, p-I*κ*B*α*, and I*κ*B*α* were analyzed using Western blotting. (b–d) Quantification of the relative density of p-NF-*κ*B/NF-*κ*B, p-IKK*α*/IKK*α*, and p-I*κ*B*α*/I*κ*B*α*. Data are reported as the means ± SD; *n* = 4. ^##^*p* < 0.01 compared with the control group; ^∗∗^*p* < 0.01 and ^∗^*p* < 0.05 compared with the OVA group.

**Figure 8:**
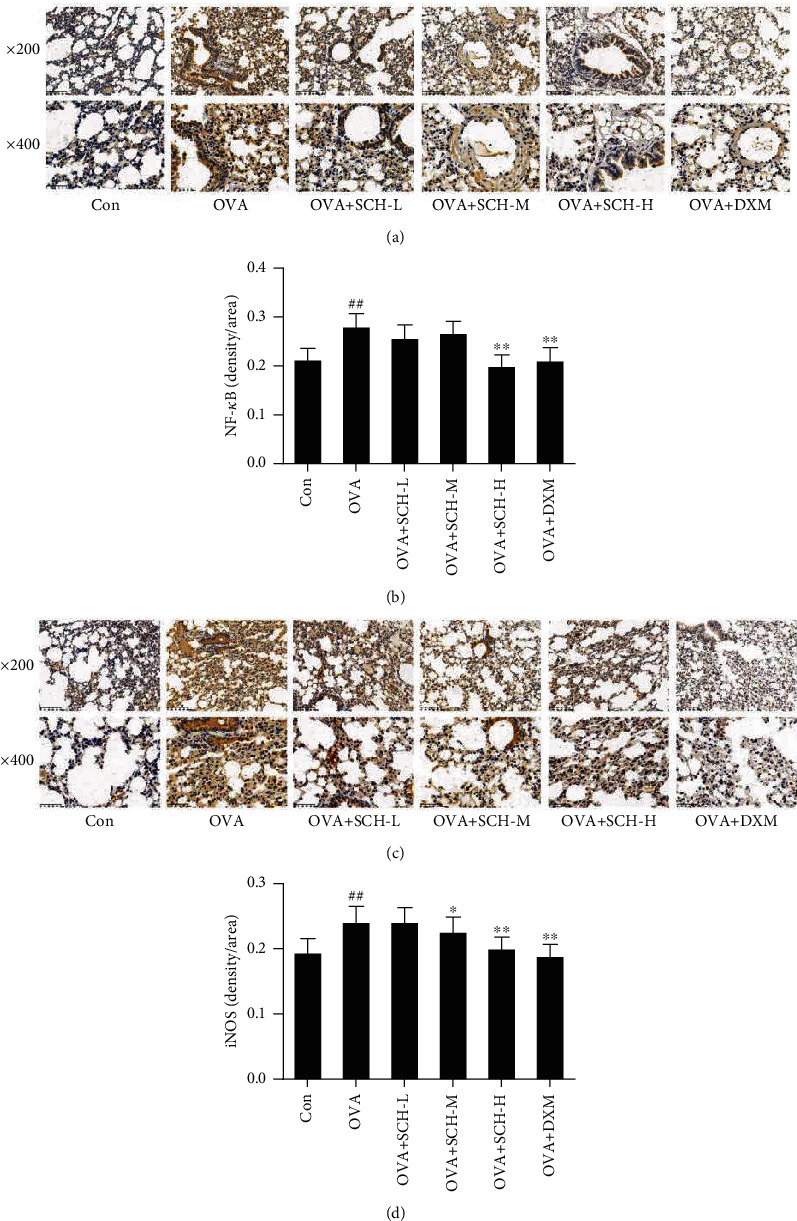
Effect of SCH on the levels of NF-*κ*B and iNOS. (a and c) The levels of NF-*κ*B and iNOS in lung tissues were detected using immunohistochemistry. (b and d) Quantification of the average density of NF-*κ*B and iNOS. At ×200 magnification, scale bars represent 100 *μ*m. At ×400 magnification, scale bars represent 50 *μ*m. Data are presented as the means ± SD; *n* = 6. ^##^*p* < 0.01 compared with the control group; ^∗∗^*p* < 0.01 and ^∗^*p* < 0.05 compared with the OVA group.

The effect on the NF-*κ*B pathway was explored in this study to further determine the underlying mechanism by which SCH eases asthma. OVA-induced injury resulted in an increase in p-NF-*κ*B and p-IKK*α* levels compared to the control group (Figures 7(b) and 7(c)). Intervention with DXM and SCH reversed these changes in NF-*κ*B and IKK*α* phosphorylation. The phosphorylation of I*κ*B*α* was also remarkably decreased in response to OVA-induced injury, and this decrease was reversed by SCH intervention (Figure 7(d)). IHC showed increased nuclear translocation of p65 and iNOS in the OVA+SCH-M, OVA+SCH-H, and OVA+DXM groups (Figures 8(b) and 8(d)).

### 3.8. SCH Activates Nrf2 Pathways

**Figure 9:**
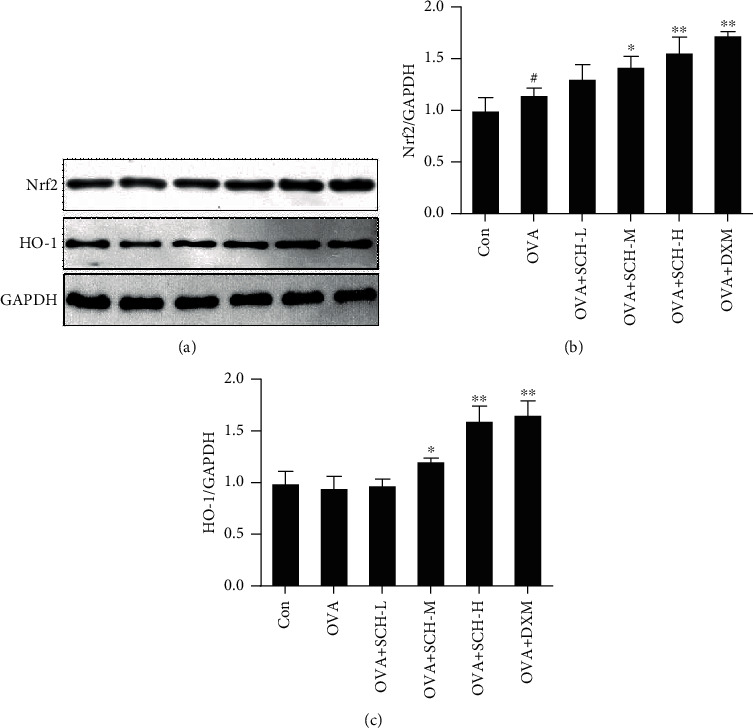
Effect of SCH on the activation of the Nrf2 pathway. (a) The expression of the HO-1 and Nrf2 proteins was analyzed using Western blotting. (b and c) Quantification of the relative density of HO-1 and Nrf2. Data are presented as the means ± SD; *n* = 4. ^#^*p* < 0.05 compared with the control group; ^∗∗^*p* < 0.01 and ^∗^*p* < 0.05 compared with the OVA group.

**Figure 10:**
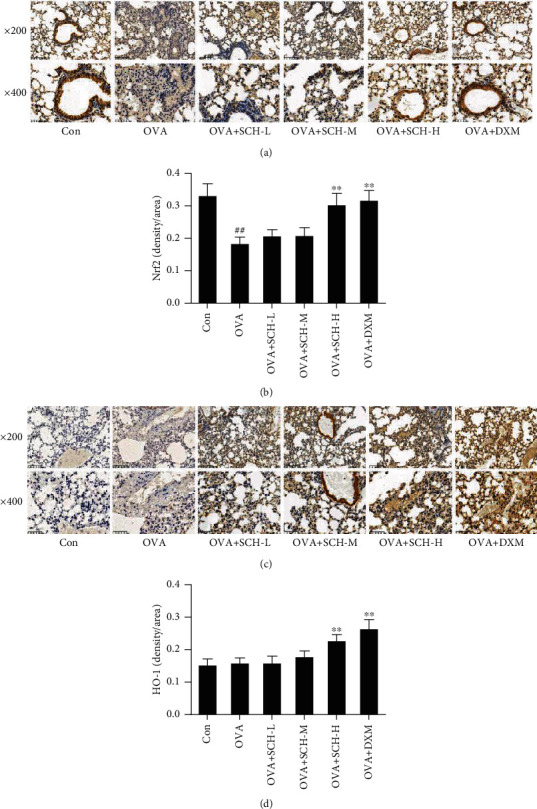
Effect of SCH on the expression of Nrf2 and HO-1. (a and c) The levels of Nrf2 and HO-1 were determined using IHC. (b and d) Quantification of the average density of Nrf2 and HO-1. Data are reported as the means ± SD; *n* = 6. ^##^*p* < 0.01 compared with the control group; ^∗∗^*p* < 0.01 compared with the OVA group.

The OVA challenge decreased the expression of Nrf2, whereas this inhibitory effect was successfully reversed in the OVA+SCH-M, OVA+SCH-H, and OVA+DXM groups (Figures 9(a) and 9(b)). DXM and SCH treatments increased the expression of Nrf2 compared to animals with OVA-induced injury. Likewise, the level of HO-1 was slightly increased in response to OVA application and additionally increased by SCH and DXM treatments (Figures 9(a) and 9(c)). These consequences were highly consistent with the outcome of IHC. OVA-induced injury remarkably increased the level of HO-1 compared to that in the controls, and an even larger increase was observed in animals treated with SCH and DXM (Figure 10).

## 4. Discussion

Asthma is a chronic and complicated airway inflammatory disease that simultaneously affects many people worldwide [5]. Asthma can cause severe morbidity and even mortality. More importantly, bronchodilators (such as *β*-agonists), steroids, and leukotriene inhibitors are the main treatment strategies for asthma. However, potential targeted mechanisms and interventions for asthma require further research [15]. Numerous studies have identified a critical role for the inflammatory response in the process of asthma, and asthma is always accompanied by airway inflammation, which is mainly characterized by inflammatory cell infiltration and increased mucus secretion [16–18].

In recent years, natural products and extracts from natural herbaceous plants have been investigated in the pharmaceutical industry due to their prominent biological activities, and herbal drugs have attracted increasing attention as effective treatments for many chronic diseases. For example, ripe hawthorn fruit provided better beneficial remediation of the cardiopulmonary circulation system due to its abundant proanthocyanidins and flavan-3-ol (-)-epicatechin levels correlated with (-)-epicatechin levels [19]. Meadowsweet likewise protects the digestive tract mucosa by reducing excess acidity and relieving nausea [20]. In the present study, SCH extracted from Schisandra appeared to contribute to ameliorating the airway inflammatory response in OVA-induced asthmatic mice, indicating its potential role in clinical asthma treatment.

The anti-inflammatory activity of extracts from natural herbaceous plants toward asthma has been widely studied. For example, forsythiaside A decreases allergic airway inflammation caused by OVA by activating the Nrf2 pathway [16]. Aloperine attenuates the inflammatory reaction in the asthmatic lung by decreasing inflammatory cell infiltration and preventing the accumulation of inflammatory factors in the airways [3], and SCH reduces allergic airway inflammation caused by OVA through the activation of the Nrf2 pathway [21]. In this study, we suggested the phylactic effect of SCH on an allergic airway inflammation model induced by OVA, which was mediated by its anti-inflammatory and antioxidant effect.

Asthma is characterized by the activation of Th2 cell activity, and the production of Th2-type cytokines results in the Th2-dominated immune response. IL-13, IL-5, and IL-4 are the primary cytokines released by Th2 cells. Therefore, increased levels of IL-13, IL-5, and IL-4 mediate the development of asthma [21]. The production of IgE is related to allergic reactions, such as allergic asthma. The production of allergen-specific IgE is mediated by activating the inflammatory response and mast cells [22]. In the present study, SCH specifically reduced the numbers of neutrophils, lymphocytes, macrophages, and eosinophils during allergic airway inflammation caused by OVA, accompanied by a decrease in the levels of proinflammatory factors, such as AHR and IgE, and lower expression of KC. These helpful effects of SCH are consistent with the results of the histopathological examination, indicating a decrease in airway inflammation and mucus production. All these results revealed the antiasthmatic effects of SCH.

As the vital calibrator of cytobeneficial proteins, the transcription factor Nrf2 decreases AHR, airway inflammation, secretion of Th2 cytokines, and mucus teratogenesis [23]. Moreover, Nrf2 signaling is a key factor determining susceptibility to allergic asthma, and Nrf2 reduces the wheezing phenotypes of asthma model mice [8]. In addition, HO-1 effectively reduces the airway inflammatory reaction and the secretion of mucus in individuals with asthma [8]. The effect of SCH on the Nrf2 signaling pathway was detected to further study its protective mechanism. Based on our results, SCH reduced allergic airway inflammation caused by OVA by activating the Nrf2 pathway. Oxidative stress is a vital characteristic of the etiopathogenesis of asthma. ROS are mainly generated by neutrophil alkaline phosphatase [24]. Furthermore, ROS may cause DNA damage and lipid peroxidation. In contrast, GSH-Px is a crucial marker of oxidative stress that plays a vital role in maintaining the integrity of the epithelium. In the present study, SCH meaningfully restrained oxidative stress caused by OVA, which was verified by the reduced MDA content and increased CAT, GSH-Px, and SOD contents. All of these consequences indicate that SCH protects against OVA-induced asthma by eliminating inflammation and oxidation.

Activation of the NF-*κ*B pathway in individuals with asthma effectively enhances inflammatory damage around the airway induced by antigens, which is an effective contributor to the adaptive immune response [25]. Specifically, when inflammatory cytokines generate activating effects, I*κ*B*α* bound to NF-*κ*B is rapidly phosphorylated by I*κ*B*α* kinase, and then, p-I*κ*B*α* is degraded by the proteasome. The independent dimer of NF-*κ*B released by the degradation of I*κ*B*α* is transported to the cell nucleus and causes the transcription of miscellaneous target genes encoding a variety of inflammatory factors, which is related to the etiopathogenesis of asthma [26]. In particular, the upregulation of iNOS, a downstream target of NF-*κ*B, facilitates inflammation by increasing the generation of inflammatory transmitters, including inflammatory cytokines, chemokines, and oxidative stress-related factors [27]. The level of iNOS is increased in patients with asthma, along with the upregulation of NF-*κ*B [28]. The production of iNOS during the development of asthma results in NO generation, which further exacerbates asthma lesions because NO represents a destructive factor related to oxidative stress [29]. Hence, inhibiting the NF-*κ*B/iNOS pathway is considered an important treatment of asthma. According to our experimental data, SCH dramatically inhibited the expression of NF-*κ*B and iNOS in asthmatic mice in a dose-dependent manner. Moreover, SCH inhibited the degradation of p-I*κ*B*α*, which is essential for NF-*κ*B activation, in a dose-dependent manner. Our results suggested that SCH may alleviate allergic airway inflammation by inhibiting the NF-*κ*B pathway, similar to a previous study [30].

Taken together, we found that SCH obviously reduced the infiltration of inflammatory cells in asthmatic mice. In terms of the mechanism, SCH alleviates asthma by promoting the production of Nrf2 and HO-1, while reducing the levels of NF-*κ*B and IKK*α* and increasing the level of I*κ*B*α*, indicating that SCH inhibits airway inflammation by restraining NF-*κ*B but promoting the activation of the Nrf2 pathway. The present study will provide new strategies for the further clinical treatment of asthma.

## Data Availability

All data generated or analyzed during this study are included in this article.
